# Supplementary material for: Adaptation to chronic ER stress enforces pancreatic β-cell plasticity
Source: Nat Commun. 2022 Aug 8;13:4621. doi: 10.1038/s41467-022-32425-7 (PMC9360004; doi:10.1038/s41467-022-32425-7)
Supplement: Supplementary file 1 — Supplementary Information [file 41467_2022_32425_MOESM1_ESM.pdf]

Supplementary Information for

Adaptation to chronic ER stress enforces pancreatic β-cell plasticity

By Chen *et al.* 2022

Supplementary Figure 1.

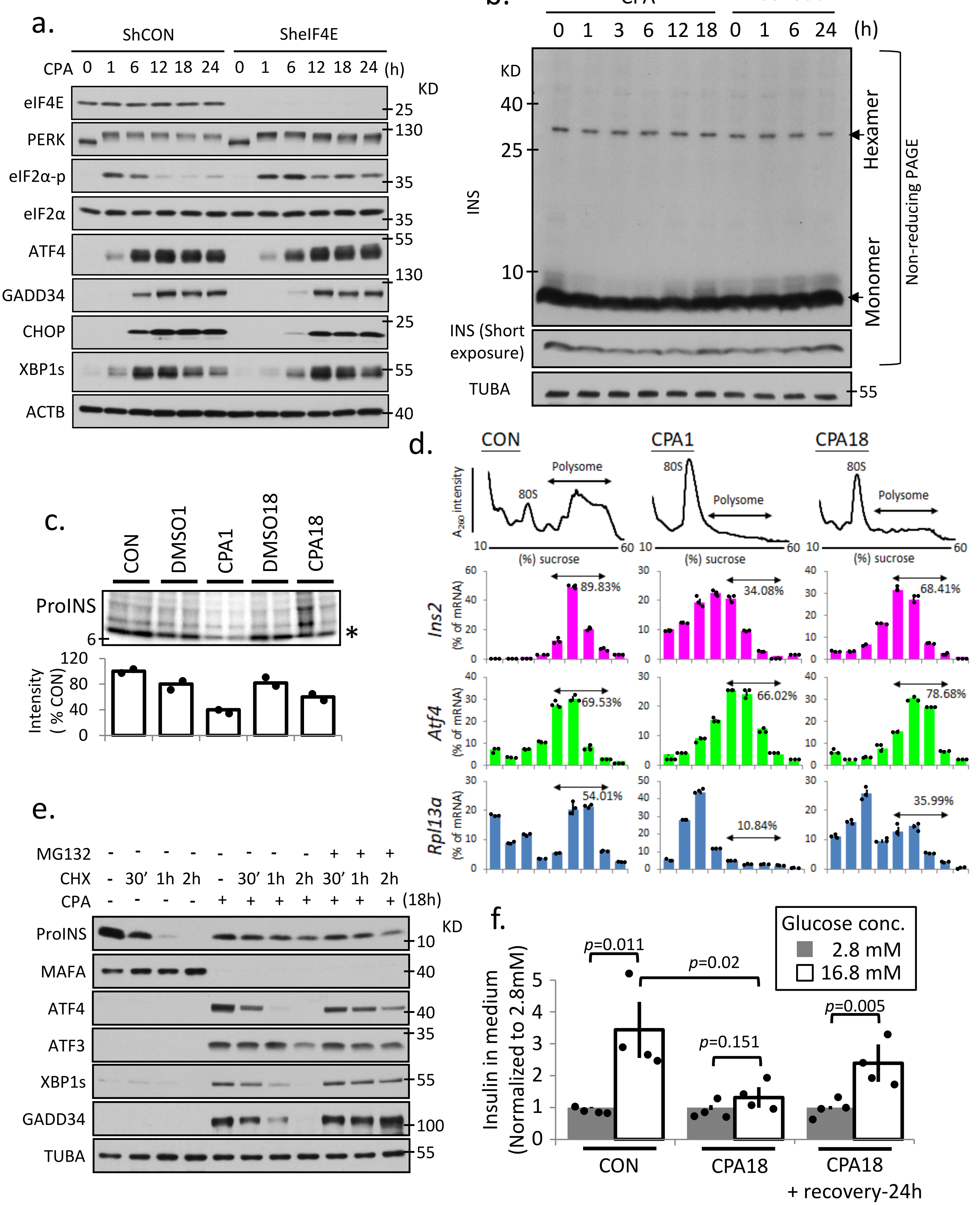

Supplementary Figure 1. Dynamic regulation of proinsulin synthesis during in MIN6 cells treated with CPA. a and **e** Western blot analysis of the indicated proteins in MIN6 cells expressing shRNAs for CON or the eIF4E mRNA and treated with CPA for the indicated times (**a**) or treated with the protein synthesis inhibitor CHX or the inhibitor of the proteosomal activity, MG132, in the presence or absence of CPA for the indicated times (**e**). **b** Western blot analysis of insulin by non-reducing SDS-PAGE electrophoresis. Arrows indicated either insulin monomer or hexamer. **c** SDS-Page analysis of metabolically labeled proinsulin in MIN6 cells treated with CPA for the indicated times. *.Quantification of two independent experiments is shown. Source data are provided as a Source Data file. **d** Distribution of the indicated mRNAs on polysome profiles of cell extracts isolated from MIN6 cells treated with CPA for the indicated times. **f** Normalized to each condition of treatment evaluation of GSIS in MIN6 cells treated for the indicated times with CPA, *n*=4 independent experiments. Error bars represent S.E.M. *p*-value represents the statistical test by two-tailed Student's *t*-test. Dots in all plots represent independent experiments. Source data are provided as a Source Data file.

Supplementary Figure 2.

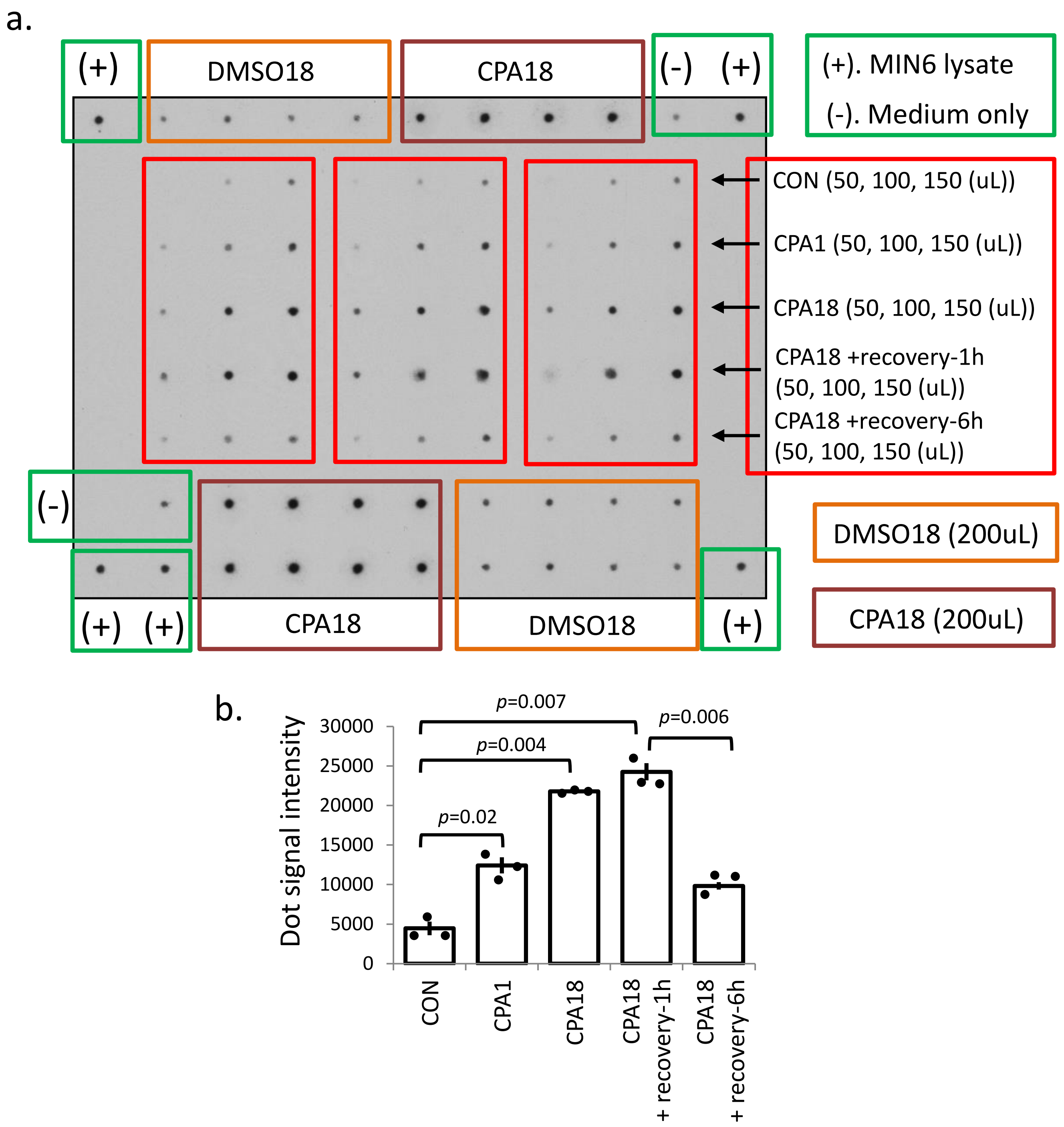

Supplementary Figure 2. Evaluation of secreted proinsulin from MIN6 cells treated with CPA. **a** Dot blot analysis of proinsulin secreted in the media from MIN6 cells treated with CPA for 1h or 18h, or washout of CPA following 18h treatment, for 1h or 6h. (+): indicates MIN6 cell lysates and serves as a positive control for proinsulin. (-): indicates growth medium not applied to MIN6 cells and serves as a negative control. Red boxes: indicate in a vertical direction the treatment and times of treatment and in a horizontal direction the increasing concentration of the medium volume (50, 100, 150 μl), collected from MIN6 cells incubated at the indicated times. Three independent experiments are shown. Orange boxes: indicate medium collected from MIN6 cells treated with the vehicle of CPA, DMSO, for 18h (200 μl). Purple boxes: indicate the medium collected from MIN6 cells treated with CPA, for 18h (200 μl). Three independent experiments and four technical replicates for each independent experiments are shown. **b** Quantification of the dot-signal intensity in A, from 3 independent experiments of MIN6 cells treated with CPA for the indicated times. Error bars represent S.E.M. *p*-value represents the statistical test by two-tailed paired Student's *t*-test. Dots in all plots represent independent experiments. Source data are provided as a Source Data file.

Supplementary Figure 3.

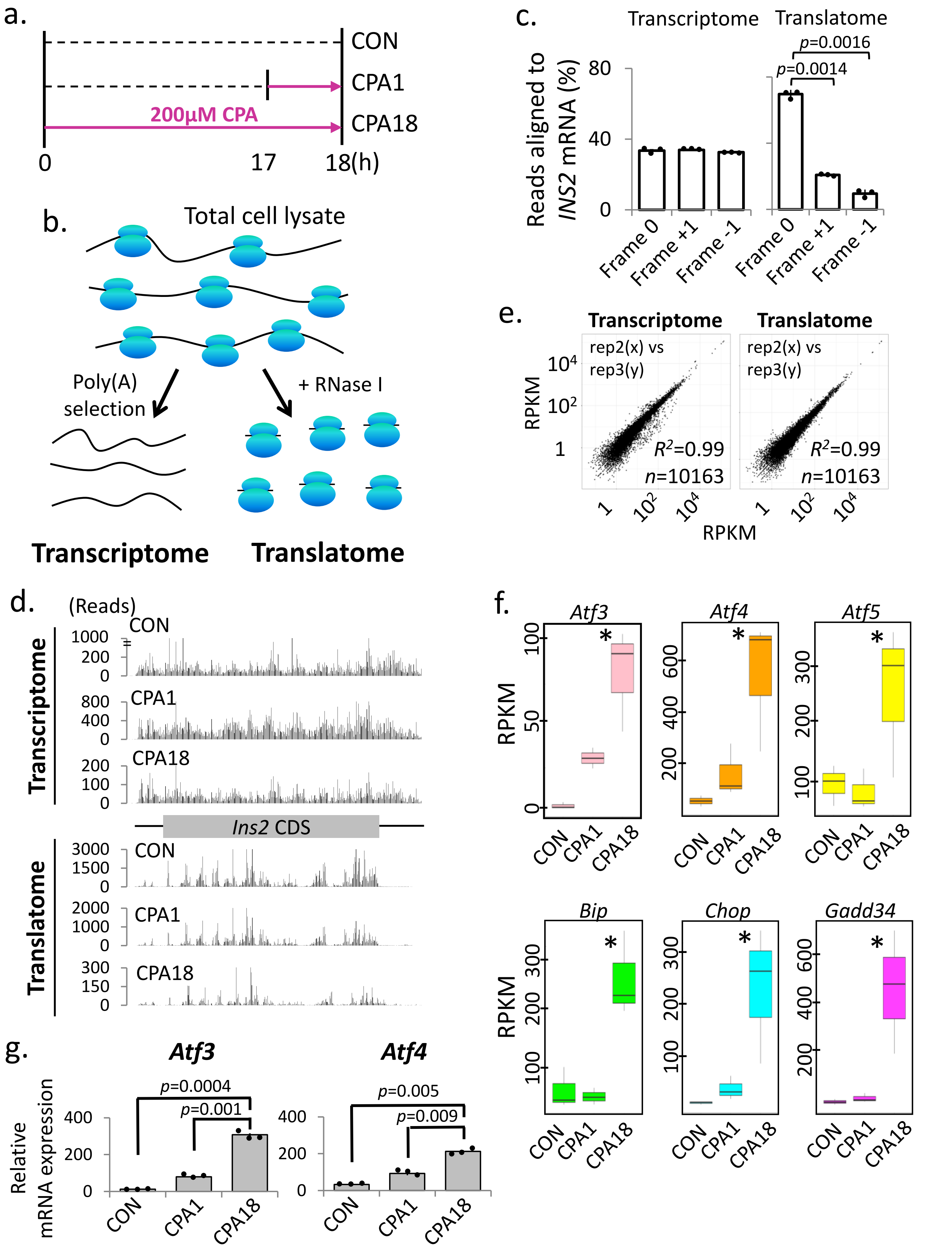

Supplementary Figure 3. Quality controls and validations of the ribosome foot printing and RNA-seq datasets. **a**-**b** Overview of experimental setup. Transcriptomes were generated from poly(A) selected RNA and translatomes from ribosome protected RNA fragments. **c**-**d** Reading frame analysis (**c**) and RNA sequencing reads distribution (**d**) across the *Ins*2 mRNA. **e** Scatterplots represent the reproducibility of transcriptomes and translatomes in two independent experiments. **f** Gene expressions changes in the transcriptomics dataset from MIN6 cells treated as in (**a**). The boxes indicate the 25–75th percentiles, the midline indicates the median, whiskers show the maximum/minimum values. *$p<0.0001$, two-sided Wilcoxon–Mann–Whitney test. **g** Relative expression levels for the indicated mRNAs, measured by qRT-PCR. $n=3$ independent experiments. Error bars represent S.E.M. p-value represents the statistical test by two-tailed paired Student’s t-test. Source data are provided as a Source Data file.

Supplementary Figure 4.

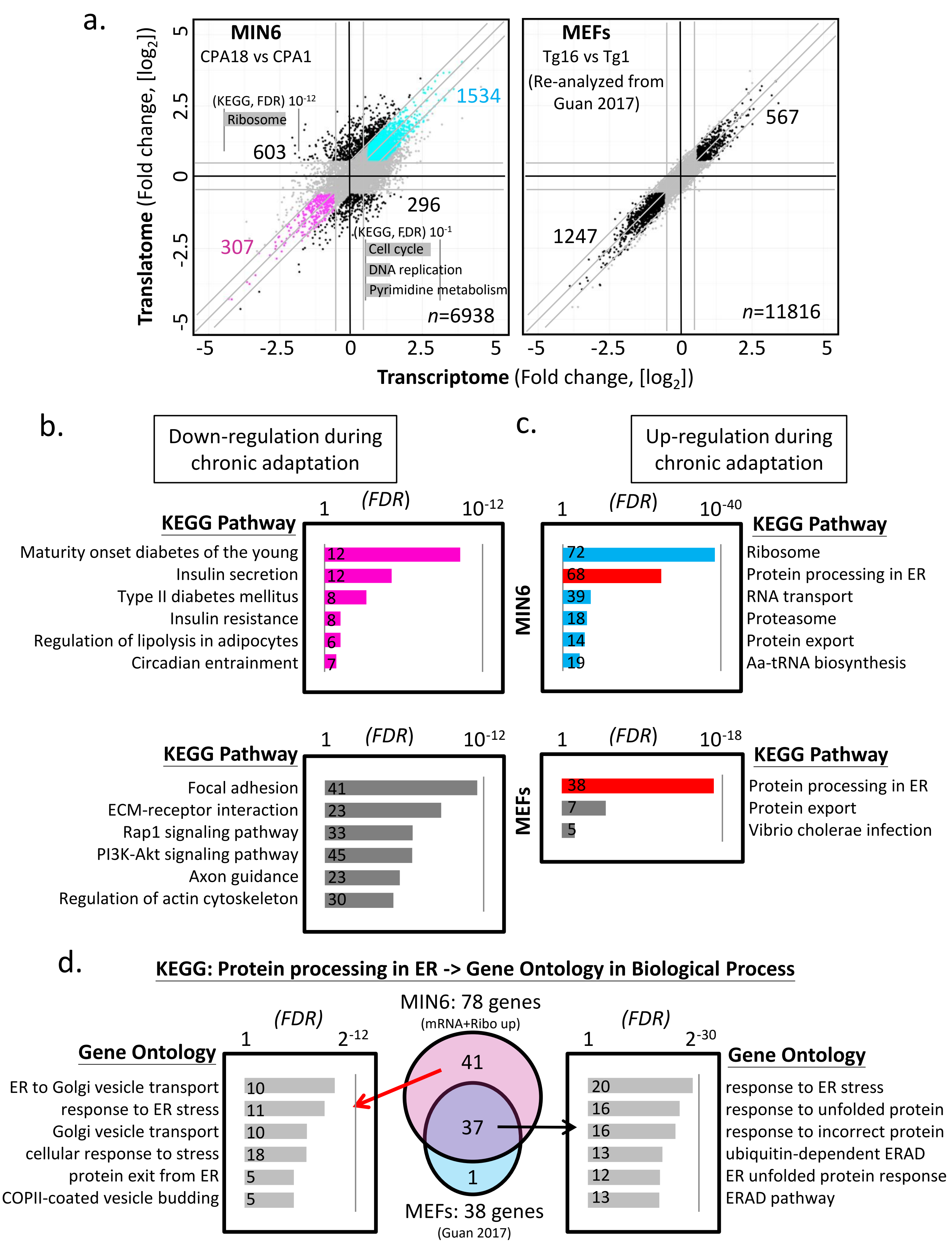

Supplementary Figure 4. Comparative analysis of transcriptomes and translatomes between MIN6 and MEFs in response to ER stress. **a** Scatterplots of fold changes in MIN6 (*left)*, and MEFS (*right*), during progression from acute to chronic ER stress (CPA18 vs CPA1 and Tg16 vs Tg1, respectively). KEGG pathway analysis of genes with either decreased mRNA abundance **b** or increased mRNA abundance (c) in MIN6 (*upper)* and MEFs (*lower)* panels. (d) Gene ontology analysis for genes in the KEEG pathway “protein processing in ER” in MIN6 and MEFs. Notice, MIN6 cells have 68 genes with increased mRNA abundance (panel C, upper) and 10 genes with increased ribo^{ocp} (panel A, included in the 603 genes).

Supplementary Figure 5.

a.

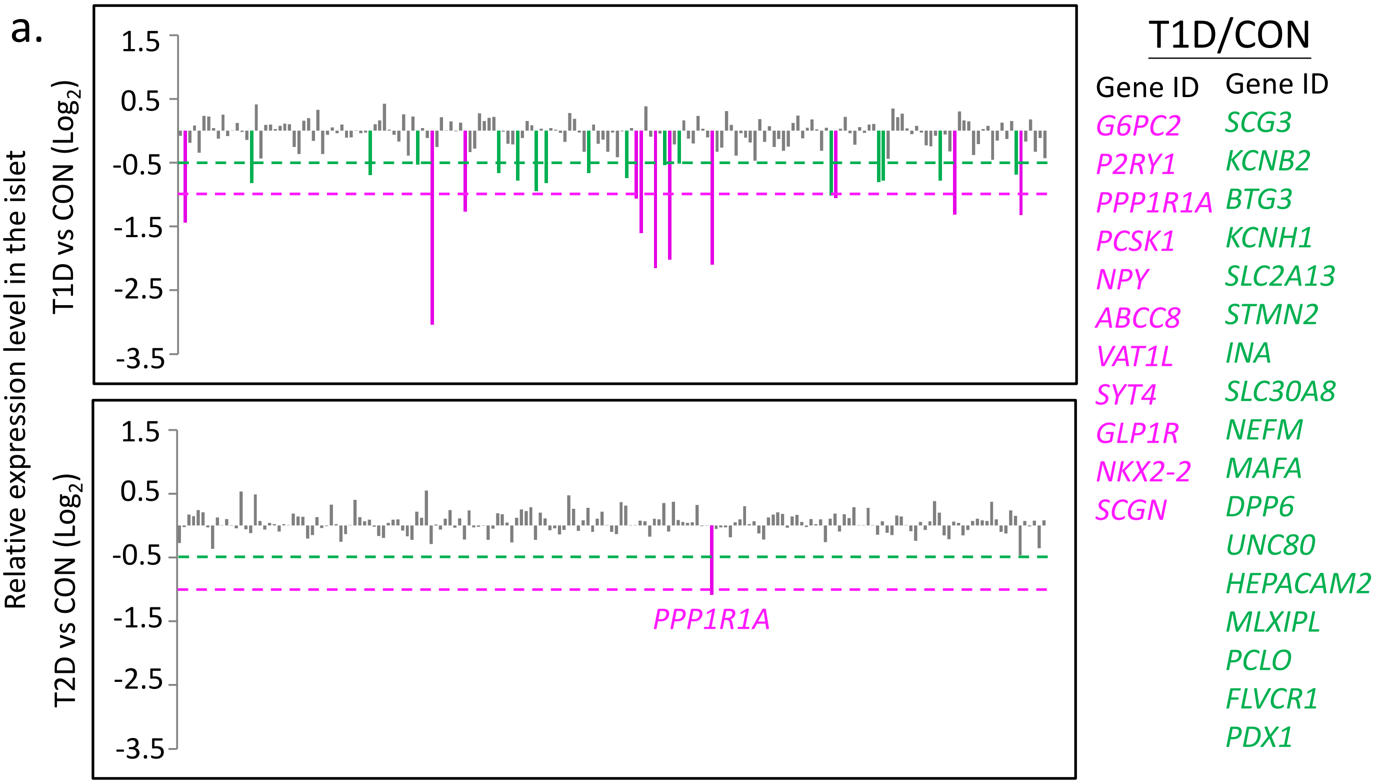

b.

PancDB No.	Age	Sex	T1D duration	α cell (%)	β cell (%)	δ cell (%)	Pp cell (%)	Acinar cell (%)	Duct cell (%)	Others (%)
HPAP019	24	M	ND	874 (21.51)	1278 (31.45)	26 (0.64)	822 (20.23)	613 (15.08)	249 (6.13)	202 (4.97)
HPAP022	37	F	ND	511 (13.74)	1069 (28.75)	70 (1.88)	73 (1.96)	380 (10.22)	1097 (29.51)	518 (13.93)
HPAP024	18	M	ND	126 (6.78)	1106 (59.59)	278 (14.98)	50 (2.69)	165 (8.89)	24 (1.29)	107 (5.77)
HPAP026	24	M	ND	200 (35.53)	94 (16.7)	60 (10.66)	37 (6.57)	66 (11.72)	49 (8.7)	57 (10.12)
HPAP029	23	M	ND	440 (19.51)	423 (18.76)	352 (15.61)	75 (3.33)	192 (8.51)	406 (18)	367 (16.27)
HPAP020	14	M	5 days	4319 (49.98)	1055 (12.21)	116 (1.34)	62 (0.72)	2229 (25.8)	629 (7.28)	231 (2.67)
HPAP021	13	F	7 years	1526 (32.18)	812 (17.12)	44 (0.93)	0 (0)	1780 (37.54)	509 (10.73)	71 (1.5)
HPAP023	17	F	7 years	300 (34.92)	86 (10.01)	10 (1.16)	1 (0.12)	215 (25.03)	171 (19.91)	76 (8.85)
HPAP028	4	M	3 years	861 (20.59)	1407 (33.64)	394 (9.42)	31 (0.74)	365 (8.73)	735 (17.58)	389 (9.3)
HPAP032	10	F	3 years	538 (22.63)	942 (39.67)	468 (19.69)	0 (0)	217 (9.13)	47 (1.98)	165 (6.94)
HPAP055	24	M	7 years	58 (3.25)	77 (4.32)	793 (44.5)	2 (0.11)	77 (4.32)	569 (31.93)	206 (11.56)

c.

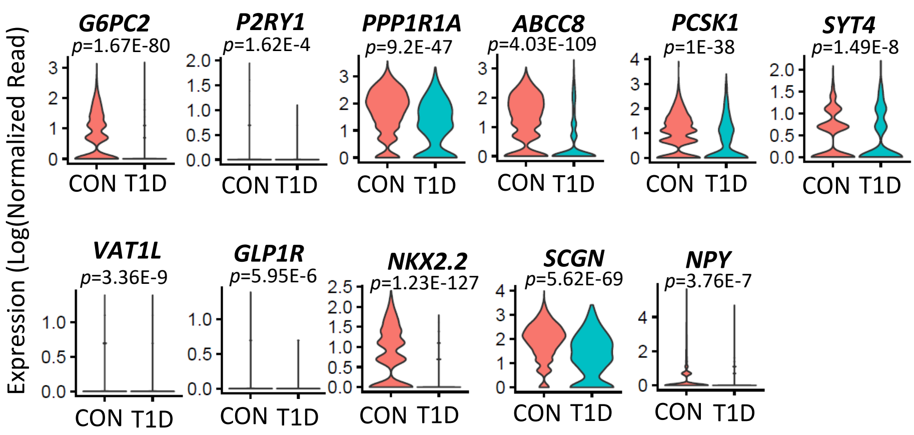

Supplementary Figure 5. β-cell ER stress 334 gene set expression in T1D/T2D datasets. **a** Relative expression of genes in T1D (*upper*), and T2D (*lower*), islet microarray datasets. Genes showing higher decrease in expression (magenta) ($\log_2 < 1$, T1D or T2D vs CON) or moderate decrease in expression (green) ($0.5 < \log_2 \leqq 1$, T1D or T2D vs CON) are highlighted. **b** Description of healthy donors and T1D patients who donated islets for the scRNA-seq analysis. **c** violin-plots of genes in β-cells scRNA-seq datasets. *p*-value represents the statistical test by two-sided Wilcoxon–Mann–Whitney test.

Supplementary Figure 6.

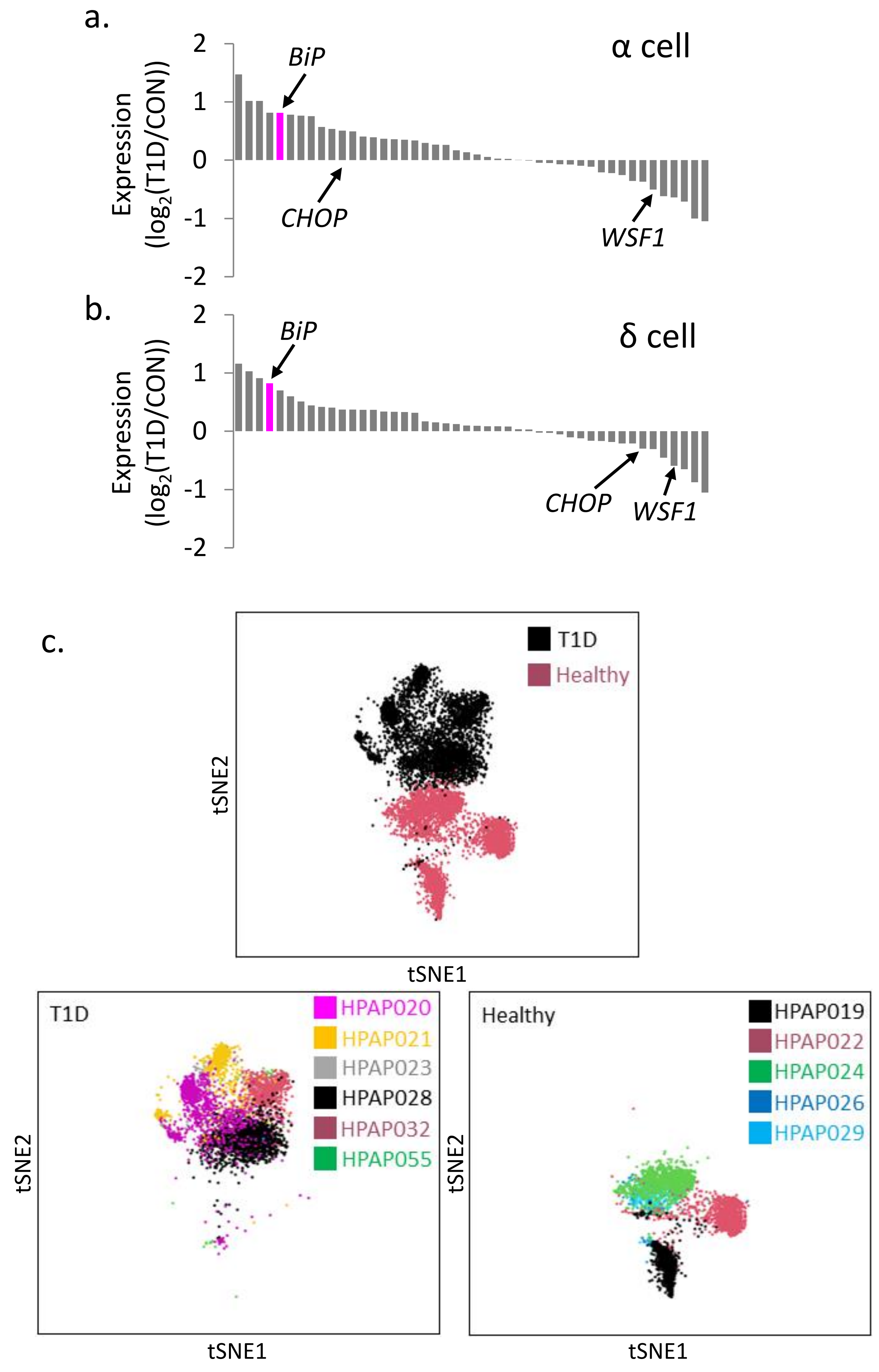

Supplementary Figure 6. Differential gene expression and clustering of scRNA-seq data from healthy and T1D subjects. **a**, **b** Schematic representation of genes as in Fig. 7a (right), in scRNA-seq from α-cells (**a**) and δ-cells (**b**) from healthy and T1D patients. **c** Similar analysis to Fig. 7b, with color-coded individuals from Sup. Fig. 5b. Data shown in Fig. 7b were obtained from scRNA-se data of 11 subjects. In Sup. Fig. 6c each subject is shown with a different color.

Supplementary Figure 7.

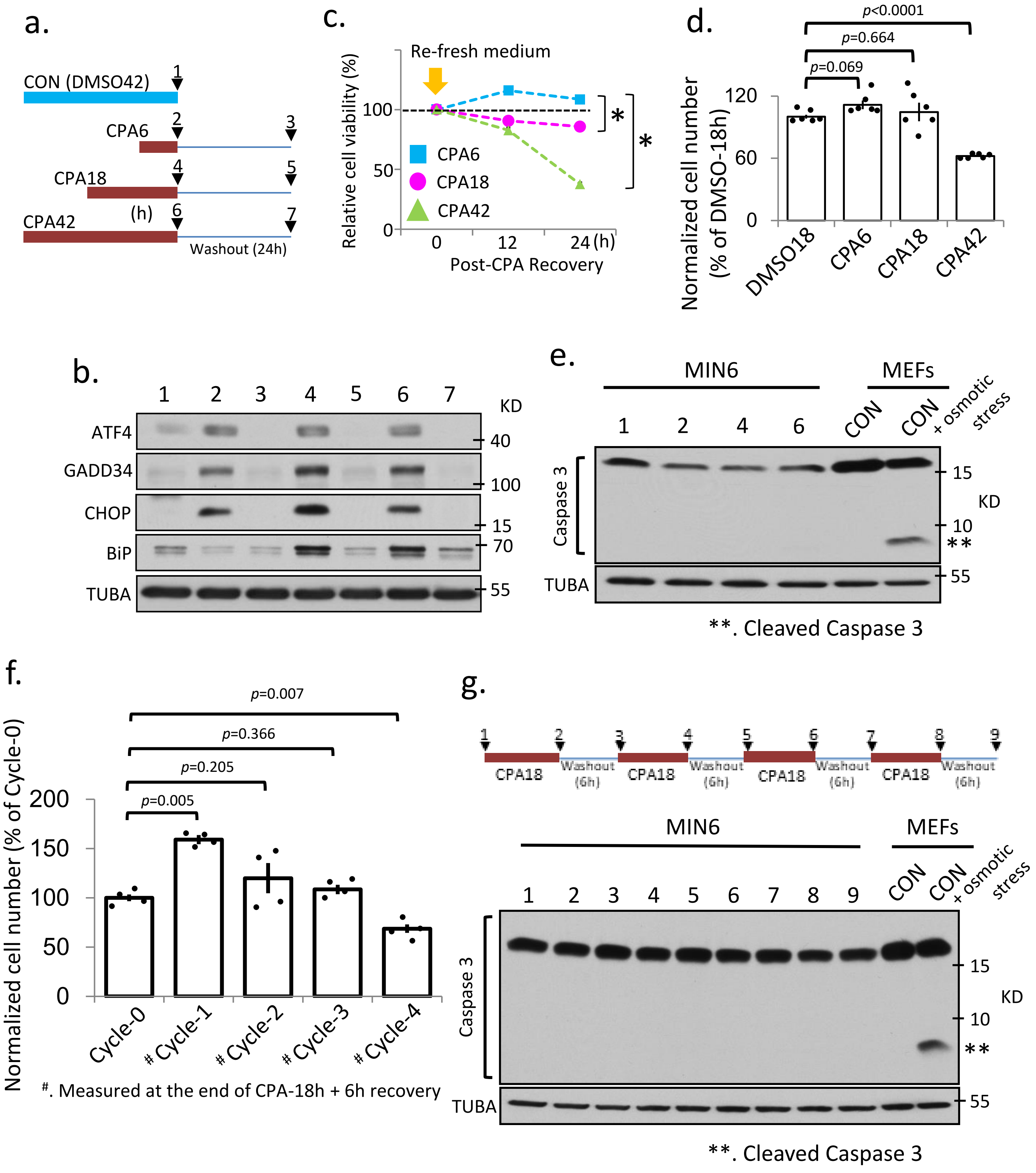

Supplementary Figure 7. MIN6 cells can recover even after prolonged ER stress exposure. **a** Experimental setup. **b**, **e**, **g** Western blot analysis for the indicated proteins and treatment of MIN6 cells as shown in **a** (**b**, **e**) or in the schematic of **g**. **c** cell viability during recovery from short (6h), chronic (18h) and prolonged (42h), ER stress exposure. **d, f** Evaluation of the number of MIN6 cells remaining attached on the culture plates following the indicated treatment with CPA. *n*=6/4 in d/f, respectively. Asterisks (**) indicate cleaved Caspase 3. As a positive control for Caspase 3 cleavage, we used cell extracts from MEFs treated with hypertonic stress 600 mOsm (Farabaugh *et al*., 2020). Error bars represent S.E.M. *$p<0.05$. *p*-value represents the statistical test by two-tailed Student's *t*-test. Dots in all plots represent independent experiments. Source data are provided as a Source Data file.

Supplementary Figure 8.

a.

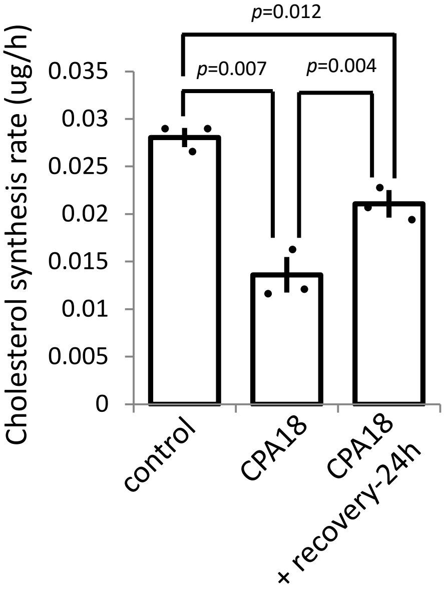

b.

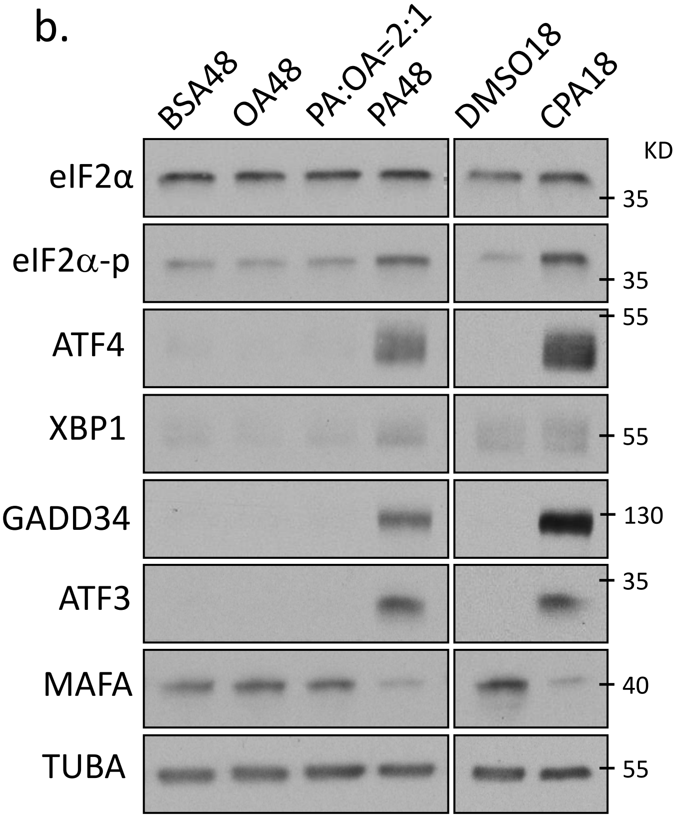

c.

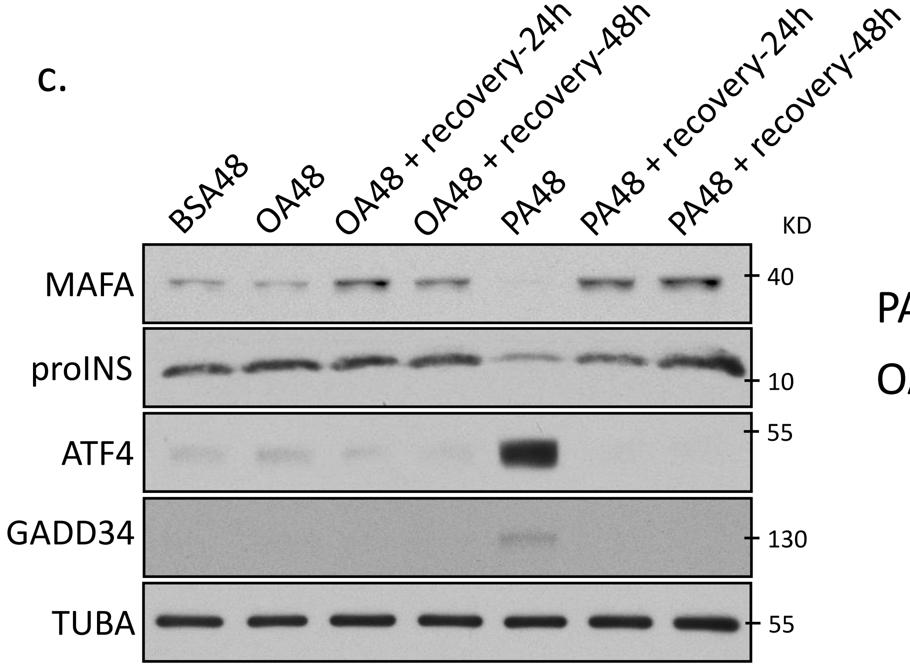

PA: Palmitic acid

OA: Oleic acid

Supplementary Figure 8. Cholesterol biosynthesis rates in MIN6 cells treated with CPA and reversible UPR gene expression in palmitic acid-treated MIN6 cells. a Cholesterol biosynthesis was measure by metabolic labeling of the cells with deuterium and analysis of labelled cholesterol by Gas Chromatography-Tandem Mass Spectrometry. *n*=3 independent experiments. Dots in all plots represent independent experiments. Source data are provided as a Source Data file. Error bars represent S.E.M. *p*-value represents the statistical test by two-tailed paired Students' *t*-test. Treatments with CPA are indicated. **b**, **c** Western blot analysis for the indicated proteins in MIN6 cells treated with oleic acid (400 mM), palmitic acid (400 mM) or a 2:1 ration of palmitic acid:oleic acid (400 mM) for 48 h, or washout after 48h treatment with palmitic acid. Analysis of extracts isolated from CPA-treated MIN6 cells is also shown.

Supplementary Figure 9.

a.

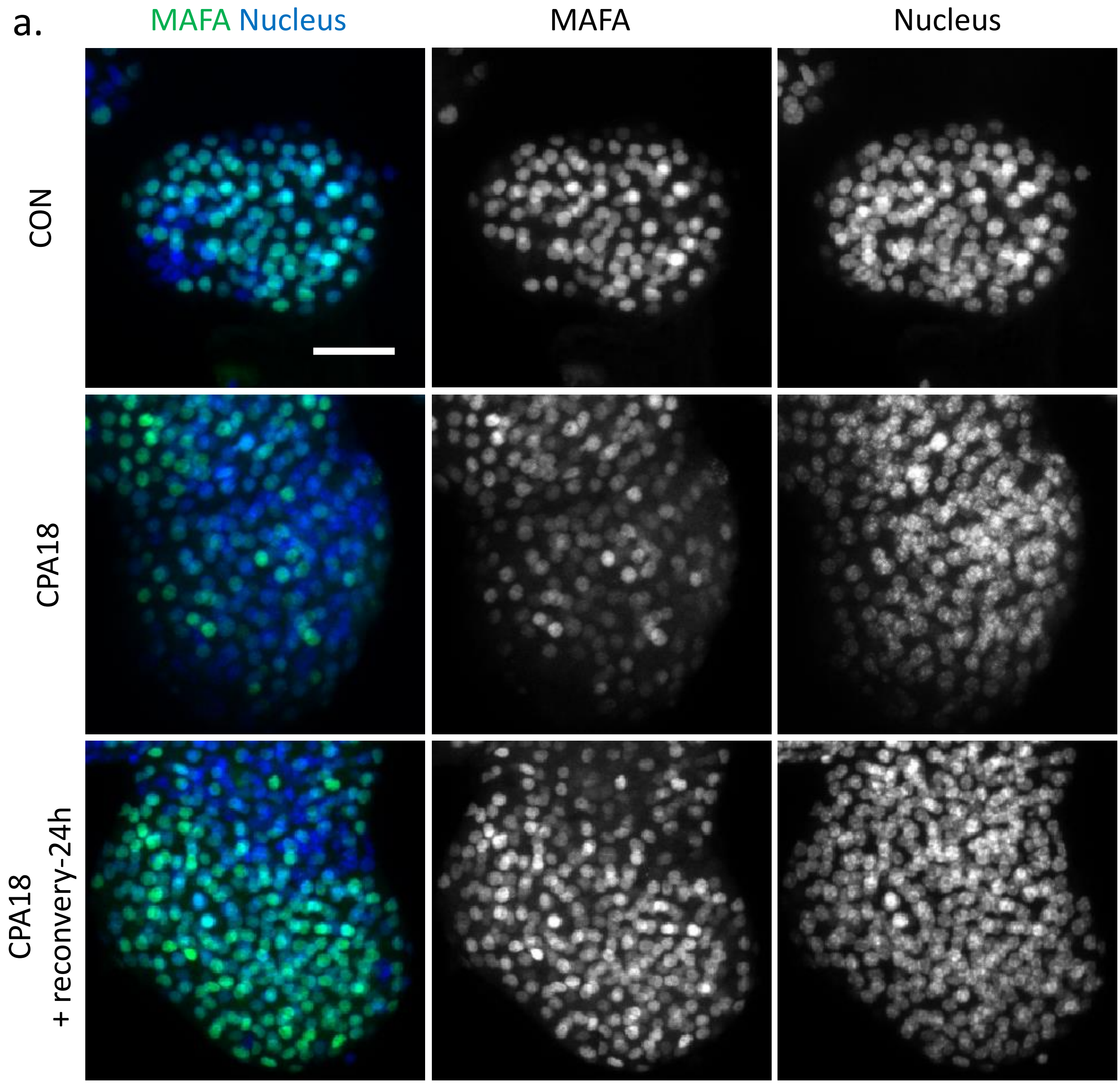

b.

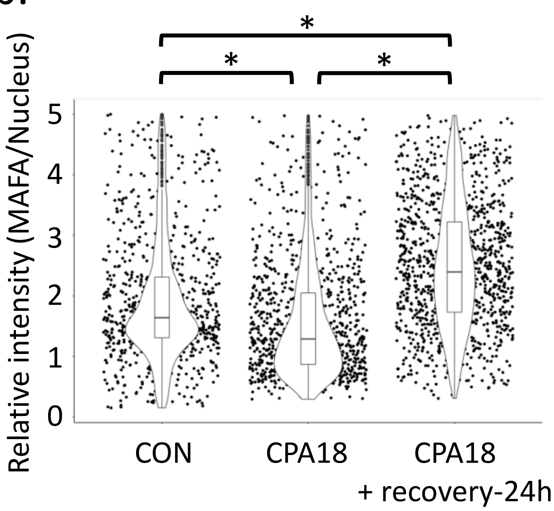

c.

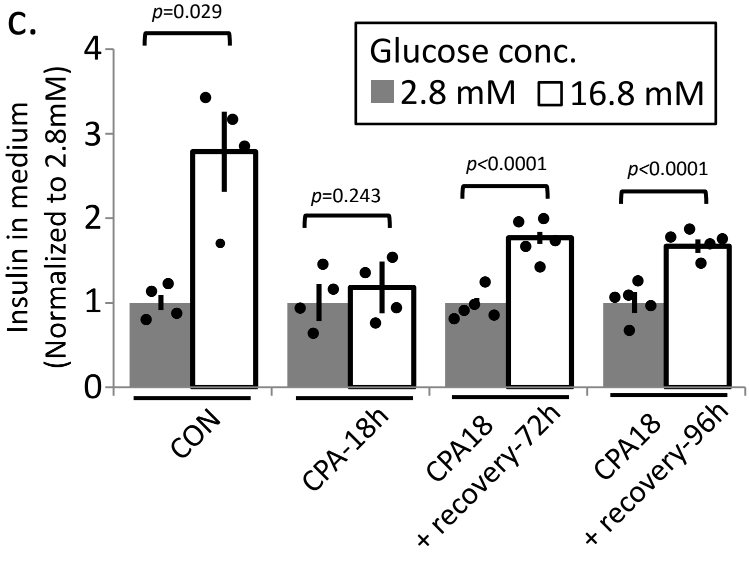

Supplementary Figure 9. Reversible CPA-induced inhibition of MAFA and GSIS in mouse islets. a Fluorescence immunocytochemistry for MAFA of mouse islets treated with CPA (200uM) for 18h and a 24h washout after 18h CPA-treatment. Scale bar, 50μm. Representative images are shown, **b** Quantification of the relative fluorescent intensity of CON (*n*=810), CPA18 (*n*=1890) and CPA18 with a 24h washout of CPA (*n*=808). $^*p<0.0001$, two-sided Wilcoxon–Mann–Whitney test. The boxes indicate the 25–75th percentiles, the midline indicates the median, whiskers show the maximum/minimum values. **c** Normalized to each condition of treatment evaluation of GSIS in mouse islets treated with CPA for 18h (*n*=4 mice), or CPA-treatment for 18h followed by washout for 72h (*n*=5 mice) or 96h (*n*=5 mice). Error bars represent S.E.M. *p*-value represents the statistical test by two-tailed Student's *t*-test. Source data are provided as a Source Data file.
