# Supplementary material for: Adaptation to chronic ER stress enforces pancreatic β-cell plasticity
Source: Nat Commun. 2022 Aug 8;13:4621. doi: 10.1038/s41467-022-32425-7 (PMC9360004; doi:10.1038/s41467-022-32425-7)
Supplement: Supplementary file 2 — Reporting Summary [file 41467_2022_32425_MOESM2_ESM.pdf]

nature portfolio

Corresponding author(s): Maria Hatzoglou
Chien-Wen Chen
Feyza Engin

Last updated by author(s): Jul 28, 2022

Reporting Summary

Nature Portfolio wishes to improve the reproducibility of the work that we publish. This form provides structure for consistency and transparency in reporting. For further information on Nature Portfolio policies, see our Editorial Policies and the Editorial Policy Checklist.

Statistics

For all statistical analyses, confirm that the following items are present in the figure legend, table legend, main text, or Methods section.

n/a	Confirmed	
☐	☒	The exact sample size (*n*) for each experimental group/condition, given as a discrete number and unit of measurement
☐	☒	A statement on whether measurements were taken from distinct samples or whether the same sample was measured repeatedly
☐	☒	The statistical test(s) used AND whether they are one- or two-sided<br>*Only common tests should be described solely by name; describe more complex techniques in the Methods section.*
☒	☐	A description of all covariates tested
☒	☐	A description of any assumptions or corrections, such as tests of normality and adjustment for multiple comparisons
☐	☒	A full description of the statistical parameters including central tendency (e.g. means) or other basic estimates (e.g. regression coefficient) AND variation (e.g. standard deviation) or associated estimates of uncertainty (e.g. confidence intervals)
☐	☒	For null hypothesis testing, the test statistic (e.g. *F*, *t*, *r*) with confidence intervals, effect sizes, degrees of freedom and *P* value noted<br>*Give P values as exact values whenever suitable.*
☒	☐	For Bayesian analysis, information on the choice of priors and Markov chain Monte Carlo settings
☒	☐	For hierarchical and complex designs, identification of the appropriate level for tests and full reporting of outcomes
☒	☐	Estimates of effect sizes (e.g. Cohen's *d*, Pearson's *r*), indicating how they were calculated

Our web collection on statistics for biologists contains articles on many of the points above.

Software and code

Policy information about availability of computer code

Data collection

No software was used for data collection in this manuscript.

Data analysis

In house RNA-seq and Ribo-seq data from CPA treated MIN6 cells and the control were processed and analyzed by Galaxy (https://usegalaxy.org/). Single cell sequencing data of diabetes patients and healthy donors were analyzed by Seurat, UMAP, Rtsne, Monocle 3 and TSCAN packages in R language. The packages are available in https://cran.r-project.org/.
Galaxy version 21.09
fastq-filter version 1.1.5
cshl_fastx_clipper version 1.0.3
Bowtie2 version 2.4.5
bedtools version 2.30.0
R version 4.1.2
RStudio version 2022.02.2-443
Seural version 4.1.0
UMAP version 0.2.7.0
Rtsne version 0.15
Monocle 3 version 1.0.0
TSCAN version 1.32.0
dplyr version 1.0.8
ggplot2 version 3.3.5

For manuscripts utilizing custom algorithms or software that are central to the research but not yet described in published literature, software must be made available to editors and reviewers. We strongly encourage code deposition in a community repository (e.g. GitHub). See the Nature Portfolio guidelines for submitting code & software for further information.

Data

Policy information about availability of data

All manuscripts must include a data availability statement. This statement should provide the following information, where applicable:

- Accession codes, unique identifiers, or web links for publicly available datasets
- A description of any restrictions on data availability
- For clinical datasets or third party data, please ensure that the statement adheres to our policy

RNA-seq and Ribo-seq data from CPA treated MIN6 cells and the control are available in GSE174679 - Adaptation to chronic ER stress enforces pancreatic beta-cell plasticity. https://www.ncbi.nlm.nih.gov/geo/query/acc.cgi?acc=GSE174679
KEGG database: https://www.genome.jp/kegg/pathway.html
PancDB database:
https://hpap.pmacs.upenn.edu/
This manuscript used raw data acquired from nPOD (www.jdrfnpod.org) and from the Human Pancreas Analysis Program (HPAP-RRID:SCR_016202) Database (https://hpap.pmacs.upenn.edu), a Human Islet Research Network (RRID:SCR_014393) consortium. Raw data was aggregated to minimize the identifiable information before analysis.

Field-specific reporting

Please select the one below that is the best fit for your research. If you are not sure, read the appropriate sections before making your selection.

☒ Life sciences ☐ Behavioural & social sciences ☐ Ecological, evolutionary & environmental sciences

For a reference copy of the document with all sections, see nature.com/documents/nr-reporting-summary-flat.pdf

Life sciences study design

All studies must disclose on these points even when the disclosure is negative.

Sample size	All quantitative data were obtained from at least three independent experiments, except S35 labeling experiment, which was obtained from two independent experiments. The statistics was done by two-tailed paired Student's t-test.<br>All fluorescence images were obtained from at least two independent experiments.<br>RNA-seq and Ribo-seq data from CPA treated MIN6 cells and the control were performed for three independent biological repeats. Transcripts identified in all three repeats were selected for bioinformatics analysis.<br>For mouse pancreatic islet of Langerhans experiments, four mice were used as a control group and five mice were used in each experimental group. For diabetes data, six type 1 diabetes patients and five healthy donors were included.<br>Mouse experiments were obtained from at least four mice per group.<br>For Western blot experiments, representative experiments are illustrated in the manuscript.
Data exclusions	No data was excluded in this study.
Replication	To reduce batch-to-batch background effects and technical variation, RNA-seq and Ribo-seq data from CPA treated MIN6 cells and the control were performed for three independent biological replicates. all attempts at replication were successful.
Randomization	For diabetes data, type 1 diabetes patients were grouped by the clinical records. For healthy control, all the available scRNA-seq data from healthy donors before May 2020.
Blinding	The result of each experiment was collected and performed by the person in charge of the experiment. The experimental results were analyzed by C-W. C. and M.H. The summary was reviewed in the group. For mouse pancreatic islet of Langerhans experiments, mice were randomly selected in a double-blind manner.

Reporting for specific materials, systems and methods

We require information from authors about some types of materials, experimental systems and methods used in many studies. Here, indicate whether each material, system or method listed is relevant to your study. If you are not sure if a list item applies to your research, read the appropriate section before selecting a response.

Materials & experimental systems

n/a	Involved in the study	
☐	☒	Antibodies
☐	☒	Eukaryotic cell lines
☒	☐	Palaeontology and archaeology
☐	☒	Animals and other organisms
☒	☐	Human research participants
☒	☐	Clinical data
☒	☐	Dual use research of concern

Methods

n/a	Involved in the study	
☒	☐	ChIP-seq
☒	☐	Flow cytometry
☒	☐	MRI-based neuroimaging

Antibodies

Antibodies used

Rabbit anti-ATF4 (1:1000, #11815, Cell Signaling); Rabbit anti-BiP(GRP78) (1:1000, #3177, Cell Signaling); Mouse anti-CHOP (1:1000, #2895, Cell Signaling); Mouse anti-eIF2α (1:1000, sc-133227, Santa Cruz); Rabbit anti-eIF2α-phospho (Ser51) (1:1000, ab32157, Abcam); Rabbit anti-GADD34 (1:1000, sc-825, Santa Cruz); Rabbit anti-PERK (1:1000, #3192, Cell Signaling); Rabbit anti-HERPUD1 (1:2000, #26730, Cell Signaling); Mouse anti-alpha-TUBULIN (1:4000, T9026, Sigma-Aldrich); Mouse anti-Proinsulin (1:1000, NB100-73013, Novus Biologials); Rabbit anti-MAFA (1:2000, #79737, Cell Signaling); Rabbit anti-PCSK2 (1:1000, #14013, Cell Signaling); Rabbit anti-PDX1 (1:2000, #5679, Cell Signaling); Rabbit anti-NKX2.2 (1:1000, PA5-72761, Invitrogen); Guinea pig anti-p62 (SQSTM1) (1:1000, GP62-C, Progen Biotechnik); Rabbit anti-LC3(ATG8) (1:1000, NB100-2220, Novus biologicals); Guinea pig anti-Insulin (1:200, in house order to Covance); Rabbit anti-PDI(P4HB) (1:200, HPA018884, Sigma-Aldrich); Rabbit anti-ERGIC53 (1:200, E1031, Sigma-Aldrich); Goat anti-rabbit IgG Alexa-488 (1:200, A-11008, Invitrogen); Goat anti-mouse IgG Alexa-594 (1:200, A-11032, Invitrogen)

Validation

Antibodies were used according to previous reports (Guan et al. Mol. Cell, 2017) and manufacturer's instructions.

Eukaryotic cell lines

Policy information about cell lines

Cell line source(s)

Mouse insulinoma MIN6 cells were purchased from AddexBio Technologies (Catalog no.C0018008).
HEK293T cells were purchased from ATCC (Catalog no. CRL-3216).
EndoC betaH3 cells were purchased from Human Cell Design.

Authentication

The usage of Human EndoC-betaH3 cells was authenticated by Human Cell Design for three years. The usage of MIN6 and HEK293T cells were not authenticated.

Mycoplasma contamination

MIN6, EndoC-betaH3 and HEK293T cells were tested negative of mycoplasma contamination before the experiments.

Commonly misidentified lines
(See ICLAC register)

There is no commonly misidentified cell lines used in the study.

Animals and other organisms

Policy information about studies involving animals; ARRIVE guidelines recommended for reporting animal research

Laboratory animals

C57BL/6J mouse (stock no.000664) was purchased from the Jackson laboratory. The mouse experiments in the study were conducted by six-week old male C57BL/6J mouse. All mice were housed in the Animal Resource Center in Case Western Reserve School of Medicine. The mice were maintained on a normal mouse diet and a 12h/12h light/dark cycle. The temperature was 75F and the humidity was 35%.

Wild animals

There is no wild animals used in the study.

Field-collected samples

There is no field collected samples in the study.

Ethics oversight

The ethical approval from the mouse experiments in this study was obtained from the Animal Resource Center in Case Western Reserve School of Medicine. The mice protocol approval number 2014-0069.

Note that full information on the approval of the study protocol must also be provided in the manuscript.
