# Supplementary material for: Adaptation to chronic ER stress enforces pancreatic β-cell plasticity
Source: Nat Commun. 2022 Aug 8;13:4621. doi: 10.1038/s41467-022-32425-7 (PMC9360004; doi:10.1038/s41467-022-32425-7)
Supplement: Supplementary file 4 — Description of Additional Supplementary Files [file 41467_2022_32425_MOESM4_ESM.pdf]

Title: Supplementary Data 1.

Description: Gene regulation under acute and chronic ER stress.

0vs1 RNA-up, upregulation of transcriptome in CPA1/CON; 0vs1 RNA-down, downregulation of transcriptome in CPA1/CON; 0vs1 tl-up, upregulation of translatome in CPA1/CON; 0vs1 tl-down, downregulation of translatome in CPA1/CON; 0v18 RNA-up, upregulation of transcriptome in CPA18/CON; 0vs18 RNA-down, downregulation of transcriptome in CPA18/CON; 0v18 tl-up, upregulation of translatome in CPA18/CON; 0vs18 tl-down, downregulation of translatome in CPA18/CON; 1v18 RNA-up, upregulation of transcriptome in CPA18/CPA1; 1v18 RNA-down, downregulation of transcriptome in CPA18/CPA1; 1v18 tl-up, upregulation of translatome in CPA18/CPA1; 1v18 tl-down, downregulation of translatome in CPA18/CPA1. 0vs1 mRNA, ratio of mRNA-seq in CPA1/CON; 0vs1 RP, ratio of Ribo-seq in CPA1/CON; 0v18 mRNA, ratio of mRNA-seq in CPA18/CON; 0v18 RP, ratio of Ribo-seq in CPA18/CON; 1v18 mRNA, ratio of mRNA-seq in CPA18/CPA1; 1v18 RP, ratio of Ribo-seq in CPA18/CPA1.

Title: Supplementary Data 2.

Description: UPR signature (GO:0030968) used in this study.

MIN6 0vs1 mRNA, ratio of mRNA-seq in CPA1/CON; MIN6 0vs1 RP, ratio of Ribo-seq in CPA1/CON; MIN6 0v18 mRNA, ratio of mRNA-seq in CPA18/CON; MIN6 0v18 RP, ratio of Ribo-seq in CPA18/CON. #N/A, genes not identified in this study.

Title: Supplementary Data 3.

Description: Summary of βEAR gene expression in MIN6, scRNA-seq and microarray in this study.

MIN6-18v0-mRNA, gene expression in mRNA-seq in MIN6 cells in CPA18/CON; MIN6-18v0-RP, gene expression in Ribo-seq in MIN6 cells in CPA18/CON; scRNA-T1D/CON, gene expression in scRNA-seq in T1D/healthy; microarray-T1D/CON, gene expression in microarray in T1D/healthy. L.Q., low quality or missing data; N.D., not detected.

Title: Supplementary Data 4.

Description: List of existing microarray data from human subjects used in this study.

Title: Supplementary Data 5.

Description: DNA oligos used in this study.
